# Anticoagulation control, outcomes, and associated factors in long-term-care patients receiving warfarin in Africa: a systematic review

**DOI:** 10.1186/s12959-022-00416-9

**Published:** 2022-10-03

**Authors:** Tamrat Assefa Tadesse, Gobezie Temesgen Tegegne, Dejuma Yadeta, Legese Chelkaba, Teferi Gedif Fenta

**Affiliations:** 1 Department of Pharmacology and Clinical Pharmacy, School of Pharmacy, College of Health Sciences, Addis Ababa University, Addis Ababa, Ethiopia; 2 Department of Pharmaceutics and Social Pharmacy, School of Pharmacy, College of Health Sciences, Addis Ababa University, Addis Ababa, Ethiopia; 3 Departmnt of Internal Medicine, School of Medicine, College of Health Sciences, Addis Ababa University, Addis Ababa, Ethiopia

**Keywords:** Anticoagulation control, Anticoagulation outcomes, Warfarin, Long-term care, Africa

## Abstract

**Background:**

Oral anticoagulation therapy with warfarin requires frequent monitoring level of anticoagulation by the international normalized ratio (INR). In Africa, studies that explore anticoagulation control, treatment outcomes, and associated factors are reported in various ways in long-term patients receiving warfarin therapy to generate concrete scientific evidence.

**Methods:**

The literature search was conducted in PubMed, Cochrane Library, African Journal of Online databases, Google Scholar, and Google. An advanced search strategy was computed to retrieve relevant studies related to anticoagulation control and outcomes. Duplication, title and abstract screening, and full-text assessment were conducted in Covidence software. Study quality was assessed using the Joanna Briggs Institute Critical appraisal quality assessment tool. The systematic review is registered in PROSPERO (CRD42021260772) and performed based on the Preferred Reporting Items for Systematic Reviews and Meta-analysis (PRISMA) guideline.

**Results:**

Out of 298 identified articles, 18 articles were eligible for the final review and analysis. The mean of 39.4 ± 8.4% time in therapeutic range (TTR) (29.4 to 57.3%), 36.7 ± 11.5% TTR (range 25.2–49.7%) and 46% TTR (43.5–48.5%) was computed from studies that determined TTR by Rosendaal, direct and cross-section-of-the-files methods, respectively. In this review, the lowest percentage of TTR was 13.7%, while the highest was 57.3%. The highest percentage of patients (32.25%) who had TTR ≥ 65% was reported in Tunisia, but the lowest percentages were in Namibia (10%, TTR ≥ 65%) and Kenya (10.4%, TTR ≥ 70%). Most of the included studies (11 out of 18) used Rosendaal’s method while the direct method was employed by three studies. Generally, 10.4–32.3% of study participants achieved desired optimal anticoagulation level. Regarding secondary outcomes, 1.6–7.5% and 0.006–59% of patients experienced thromboembolic complications and bleeding events, respectively. Having chronic comorbidities, taking more than two drugs, and presence of medications that potentially interact with warfarin, and patient-related factors (patients aged < 50 years old, female gender, lower education level, smoking history) were the frequently reported predictors of poor anticoagulation therapy.

**Conclusions:**

Oral anticoagulation control was suboptimal in patients taking warfarin as evidenced by low TTR in Africa. Therefore, there is an urgent need for further improving oral anticoagulation management services.

**Supplementary Information:**

The online version contains supplementary material available at 10.1186/s12959-022-00416-9.

## Background

Vitamin K-dependent anticoagulants (VKAs) continue to be the principal anticoagulants for the treatment and prevention of thromboembolism [1] despite the introduction of direct-acting oral anticoagulants (DOACs) [2, 3]. It is used for the prevention and treatment of thromboembolic events (TEEs) and their complications in patients with atrial fibrillation, pulmonary embolism, deep venous thromboembolism, and valvular heart diseases [4, 5]. However, oral anticoagulation therapy with warfarin requires frequent international normalized ratio (INR) monitoring [6]. In addition, warfarin therapy is complicated by its unpredictable pharmacokinetics and dynamics features, multiple drugs and food interactions, narrow therapeutic index, and life-threatening complications due to subtherapeutic or excessively elevated INRs [7–10].

The quality of anticoagulation control with warfarin is majorly reflected by the mean individual patients spend in the therapeutic range [11, 12]. Time in therapeutic range (TTR**)** estimates the percentage of time a patient’s INR is within the desired treatment range or goal and is used as an indicator of anticoagulation control [13]. The fraction of INRs in range or the direct method, the Rosendaal linear interpolation method, and the cross section-of-the-files method were the three common methods of TTR determination [14].

To achieve the optimal clinical outcome, the TTR should be ≥ 65% [15] and, the recent European Cardiac Society (ESC) guidelines suggested TTR of ≥ 70% [16] whereby the rates of thromboembolic events/complications and major bleeding-related due to VKA are low [17]. However, various studies conducted globally reported suboptimal anticoagulation with warfarin therapy by documenting low TTRs (< 65% [13, 18–22]. The extent of anticoagulation control and outcome in patients receiving warfarin in long-term care vary in Africa as TTR ranges from 29 to 49.7% [7, 23]. Moreover, these studies reported anticoagulation control, and treatment outcomes, and associated factors inconsistently. In addition, there has been no aggregate data in patients receiving warfarin therapy to generate concrete scientific evidence in Africa. Therefore, this systematic review was conducted to summarize anticoagulation control, treatment outcomes, and associated factors in patients taking warfarin for its various indications in Africa in long-term care by synthesizing and providing robust evidence.

## Methods

### Protocol and reporting

This systematic review is registered in the International Prospective Register of Systematic Reviews (PROSPERO) with the registration number CRD42021260772. In addition, the review was prepared based on PRISMA guidelines [24].

### Data source and search strategy

The literature search was conducted in PubMed/Ovid, Cochrane Library, African Journal of Online databases (AJOL), Google Scholar, and Google from database inception to November 2021. The reference lists of all included studies were also reviewed. The search strategy used Medical Subject Heading (MeSH) and keywords; anticoagulant agents, treatment outcome, bleeding, thromboembolism, TTR, time in therapeutic range, international normalized ratio, INR, Africa, and long-term care. These keywords were combined using “AND” and/ “OR” Boolean operators. They were combined as follows: [Anticoagulant OR (anticoagulant agents) OR (agents anticoagulation) OR (anticoagulation agents) OR (anticoagulant drugs) OR (warfarin) OR (Coumadin) OR (warfarin therapy) OR (warfarin potassium) OR (warfarin sodium) OR (vitamin K antagonist) OR (oral anticoagulant)] AND [treatment outcome OR (outcome treatment) OR (patient-related outcome) OR (clinical effectiveness) OR (treatment effectiveness) OR (treatment efficacy) OR (clinical) OR (efficacy) OR (bleeding) OR (bleeding events) OR (hemorrhage) OR (hemorrhagic events) OR (stroke) OR (ischemic stroke) OR (thromboembolism) OR (thromboembolic events) OR (hospitalization) OR (emergency department visit) OR (mortality) OR (intracranial hemorrhage) OR (intracranial bleeding)] AND [international normalized ratio OR (INR)) OR (monitoring) OR (time in therapeutic range) OR (TTR)] AND [long term care OR (long-term care) OR (outpatient) OR (outpatient department) OR (cardiac clinic) OR (hematology clinic) OR (anticoagulation clinic) OR (anticoagulation management service) OR (anticoagulation management quality)] AND [Africa OR (sub-Saharan Africa) OR (Africa central) OR (Africa eastern) OR (Africa southern) OR (Africa western) OR (Africa northern) OR (low-income country) OR (developing country)] OR (middle-income country)].

### Inclusion and exclusion criteria

Observational studies that reported on warfarin use, anticoagulation control, and outcomes among adult patients in long term care in African countries (monitoring of international normalized ratio and time in therapeutic range); or warfarin therapy-related adverse outcomes among these patient groups (bleeding events, thromboembolic events, stroke (ischemic stroke), hospitalization, emergency room visit and mortality) were included. In addition, only studies published in English were considered. Animal studies, studies conducted on admitted and emergency patients, and pharmacogenomics studies were also excluded. Furthermore, studies that reported merely other anticoagulation outcomes (patients’ knowledge, adherence, satisfaction, quality of life, economic outcomes, adverse drug events other than bleeding, warfarin drug interactions) were excluded. Further, qualitative studies, review articles, unpublished works (thesis), case reports, case series, case–control studies, letters to the editor with incomplete information, author perspective, abstract proceedings, and expert opinions were excluded from the review.

### Article screening process

Articles identified from various electronic databases were exported to ENDNOTE reference software version 9 (Thomson Reuters, Stamford, CT, USA) with compatible formats. Then, they were imported to Covidence software [25] for screening, full-text analysis, and extraction. Duplicate records were identified, recorded, and removed with Covidence. Title and abstract screening were performed by the two reviewers (TAT and GTT). Three categories (yes, no, maybe) were used during the selection process. The full text of studies reported as “yes” or “maybe” during the initial screening process were evaluated based on the eligibility criteria by two authors (TAT and GTT). Any discrepancy in the screening processes was resolved by discussion.

### Data extraction

Data were extracted by TA using a standardized data abstraction format prepared in Microsoft Excel. This tool contains data related to study characteristics (country and study setting, first author, publication year, study design, population characteristics, and sample size) and the result of studies (percentage of time in therapeutic range and warfarin-related adverse effects).

### Quality assessment

Studies’ methodological quality was assessed using Joanna Briggs Institute Prevalence Critical Appraisal Tool (JBI) for cross-sectional study [26]. It is an 8-item rating scale developed for prevalence studies. Sampling, data collection, reliability, and validity of study tools, case definition, and prevalence periods were included in the tool. The rating scale was categorized as having a low risk of bias (“yes” answers to domain questions) or a high risk of bias (“no” answers to domain questions) for each article. Each study was assigned a score of 1 (Yes) or 0 (No) for each domain, and these scores were summed to provide an overall study quality score. Studies with less than 50% scores were considered as high studies. For the final risk of bias classification, disagreements between the reviewers were resolved via consensus. Two independent authors (TAT and GTT) assessed the quality of included studies. Discrepancies between the two reviewers were resolved through discussion. The mean score of 2 authors was taken for scaling studies.

### Outcome measurement

The primary outcome of the review was a time in the therapeutic range while bleeding, thromboembolic events/complications, hospitalization, emergency department visit, and mortality were the secondary outcomes. According to the criteria of International Society on Thrombosis and Haemostasis (ISTH), major bleeding is defined as fatal bleeding and/ or symptomatic bleeding in a critical area or organ such as intracranial, intraspinal, intraocular resulting in vision changes, retroperitoneal, intraarticular, pericardial, or intramuscular with compartment syndrome; and/ or bleeding causing a fall in hemoglobin level of 2 g/dL (1.24 mmol/L) or more, or leading to transfusion of two or more units of whole blood or red cells. All non-major bleeds will be considered minor bleeds. Minor bleeds will be further divided into those that are clinically relevant and those that are not [27].

### Data management and analysis

The mean and/or median percentages of TTR or percentages of TTR were extracted in all included studies. Secondary outcomes were reported by mean, percentage, or frequency. Factors contributing to primary and secondary outcomes were reported as described by studies.

## Results

### Literature identification and search findings

**Fig. 1:**
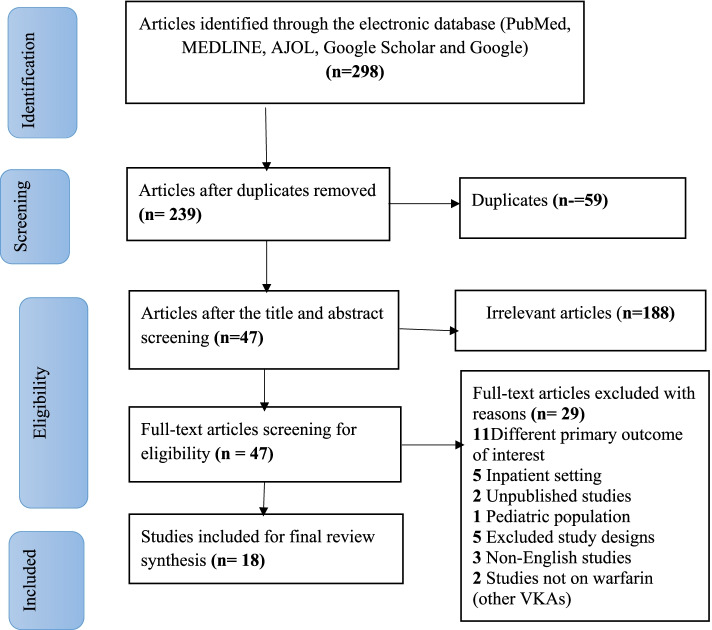
PRISMA Flow diagram for study selection for systematic review

A total of 298 articles were obtained from different electronic databases. 59 articles were removed due to duplication. Title and abstract screening were performed on 239 articles and, 188 articles were irrelevant. The full-text screening was then conducted on 47 articles, and 29 articles were excluded due to their ineligibility (e.g., absence of the outcome of interest). Finally, 18-articles were eligible and included in the systematic review (Fig. 1).

The included studies were published between 2006 to 2021. The majority of them (15 out of 18) were conducted using retrospective study designs [7, 23, 28–40]. Pre-post intervention [41] and prospective study designs [42, 43] were employed by one and two studies, respectively. Five studies were conducted in South Africa [7, 28, 33, 37, 38], 3 in Ethiopia [23, 31, 40], 2 in Sudan [32, 41], 2 in Kenya [36, 42], 2 in Tunisia [29, 43] and 2 in Botswana [39, 44]. One study was included from Namibia [35] and the remaining one study was conducted both in South Africa and Uganda. A total of 4,730 study participants were included in 18 studies. The smallest and the largest sample size was 21 [32] and 915 [45], respectively. In addition, the minimum cohort follow-up period was 4 months [46], and the maximum was 19 years [28]. All studies were conducted in outpatient settings (cardiology clinic anticoagulation clinic, INR testing clinic, warfarin clinic, cardiac, hemato-oncology, and cardiothoracic clinics, etc.). Except for one study [32], all studies were conducted in government health facilities. Various indications of warfarin were reported in the included studies (Table 1).

**Table 1:** Characteristics of included studies in the systematic review

Author ID	Country	Study design	Study population	Sample size	Follow up time	Mean or Median age in years	Service setting	Indication of warfarin
Salaheldin A, 2019 [32]	Sudan	Retrospective	Private cardiology clinic attendants	21	14 months	Mean: 64, Median: 62	Cardiology Clinic	AF, VHD, DVT, PE, LVT
Karuri et al., 2019 [36]	Kenya	Retrospective	Adult outpatients	406	2 years and 6 months	Mean: 42.7 (SD: 16.9)	Cardiac, hemato-oncology, and clinic	DVT, PE, Prosthetic valves, AF, VHD
Sana et al., 2020 [43]	Tunisia	Prospective	AF patients ≥ 20 years	915	12 months	Mean: 64.27	In- and outpatients setting	AF
Lauren et al., 2019 [35]	Namibia	Retrospective	Adult outpatients	215	12 months	Median: 46	Warfarin Clinic	DVT, PE, AF, CVA, AVR, LVT, DCM, MVR, DVR, Others
Sonuga et al., 2016 [37]	South Africa	Retrospective	Adult patients	136	6 months	Mean: 62 (for male), Median: 66 (for female)	INR Clinic	VHD, mechanical heart valve replacement
Fenta et al., 2017 [23]	Ethiopia	Retrospective	Adults outpatients	360	12 months	Mean: 35.3	Cardiac and hematology clinics	AF, DVT, PE, VHD, MI, HVR, PVR
Semakula et al., 2020 [5]	South Africa and Uganda	Retrospective	Outpatients	229	6 months	Median: 56	Anti-coagulation clinic	VTE, AF, VHD
Prinsloo et al., 2021 [38]	South Africa	Retrospective	Adult patients	191	12 months	Median: 56	INR Clinic	AF, VTE, MPHV, APS, and LVT
Ahmed et al., 2017 [41]	Sudan	Pre-and post- Intervention	Adult patients	135	12 months	Mean: 41.8	Anticoagulation clinic	MVR, DVR, total valve replacement
Botsile et al., 2020 [39]	Botswana	Retrospective	Patients aged ≥ 18 years	142	5 months	Mean: 42	INR Clinic	MHV replacement
Masresha et al., 2021 [31]	Ethiopia	Retrospective	Adult outpatients	202	2 years	Mean: 44.33	Outpatient department	AF, VHD, DVT, and PE
Kizito et al., 2016 [47]	Kenya	Prospective	Adult outpatients	147	NA	Mean: 41	Hemato-oncology and cardiothoracic clinics	Heart disease, VTE, HVR
Yimer et al., 2021[40]	Ethiopia	Retrospective	Adult outpatients	300	2 years	Mean: 56.4	Anticoagulation Clinic	AF
Sadhabiriss and Brown, 2021 [33]	South Africa	Retrospective	Adult outpatients	263	1 year	Mean age for AF patients: 64.68, mean age for PHV patients:41.83	Outpatient adult medical department	non-valvular AF, PHV, venous thrombosis or embolism, arterial or left ventricle thrombus, valvular AF, HF
Ntlokotsi et al., 2018 [28]	South Africa	Retrospective	Adult patients	95	19 years	Mean 39.7 (SD:18)	Academic hospital	HVR
Rejeb et al., 2019 [29]	Tunisia	Retrospective	Adult patients	200	3 years	Mean: 58.8 ± 12	Cardiac clinic	AF
Mwita et al., 2017 [30]	Botswana	Retrospective	Adult patients	410	2 years	Median: 46(35–58 IQR)	Outpatient medical clinic	Mechanical valves, DVT, AF, intracardiac thrombosis, pulmonary hypertension
Ebrahim et al., 2018 [7]	South Africa	Retrospective	Adult out patients	363	6 years	Median: 55(IQR 44—64)	Warfarin anticoagulation Clinic	AF, VHD, PE VTE, SLE, hypercoagulable states

*AF* Atrial fibrillation, *VHD* Valvular heart disease, *DVT* Deep vein thrombosis deep, *PE* Pulmonary embolism, *CVA* Cerebrovascular accident, *AVR* Atrial valve replacement, *LVT* Left ventricular thrombus, *DCM* Dilated cardiomyopathy, *DVR* Double valve replacement, *MVR* Mitral valve replacement, *MI* Myocardial infarction, *SLE* Systemic lupus erythematosus, *HF* Heart failure, *APS* Antiphospholipid syndrome, *MHV* Mechanical heart valve, *HVR* Heart valve replacement, *MPHV* Mechanical prosthetic heart valve, *IQR* Interquartile range, *SD* Standard deviation, *INR* International normalized ratio

### Quality assessment of included studies

With the exception of two studies, the majority of the included studies have a low-risk methodological quality according to the modified the Joanna Briggs Institute (JBI) critical appraisal tool as is indicated in a supplementary table.

### Primary outcome: time in therapeutic range

Direct, Roosendaal’s, cross-section of-the-files methods, or a mixture of direct and Roosendaal’s methods were used to determine TTR in the included studies. Eleven studies used Rosendaal’s method, while the direct method was employed by three studies. The direct method (the fraction of INRs in range) and the cross-section-of- the-files method were utilized by two studies [37, 47]. In the remaining two studies [32, 33, 35], TTR was calculated both by direct and Roosendaal’s methods [32, 35]. The included studies reported TTR as mean and /or median TTR percentages or only percentages.

The lowest percentage of TTR was 13.7% (mean) which was reported by a study conducted in adult patients with prosthetic heart valves at the medical outpatient department in KwaZulu-Natal, South Africa [33]. However, a mean TTR of 44.5% was reported in this South African study among AF patients. The highest (57.3%) was observed in a study conducted in Tunisia [29]. In another way, a higher mean TTR of 68.3% was also documented in the post-interventional study from Sudan [41]. The mean of 39.4 ± 8.4% TTR (29.4 to 57.3%), 36.7 ± 11.5% TTR (range 25.2–49.7%) and 46% TTR (43.5–48.5%) was computed from studies that determined TTR by Rosendaal, direct and cross-section-of-the-files methods, respectively.

The percentage of patients with optimal anticoagulation (TTR ≥ 65%) or above as indicated by studies was documented by 13 studies. Accordingly, the highest percentage of patients (32.25%) who had TTR ≥ 65% was reported in studies conducted in Tunisia [43] and lowest percentages i.e. 10% (TTR ≥ 65%) [35] and 10.4% (TTR ≥ 70%) [36] were obtained in studies conducted in warfarin anticoagulation clinic at Windhoek Central Hospital in Namibia and Kenyatta National Hospital (KNH), Kenya, respectively (Table 2).

**Table 2:** Anticoagulation control, primary and secondary outcomes of included studies in the systematic review

Author ID	Primary outcomes				Secondary Outcomes		
	**Method to determine TTR**	**Percentage of TTR**	**% of patients within the therapeutic range**	**% of patients with TTR ≥ 65% or indicated by a study**	**Bleeding during warfarin therapy (%)**	**Major bleeding (%)**	**Thromboembolic events (%)**
Salaheldin A, 2019 [32]	Rosendaal and Direct Method	Median 37% by Rosendaal method, and median TTR 42.9% by Direct Method	NA	23% of patients with TTR > 72%	9.50	NA	NA
Karuri et al., 2019 [36]	Rosendaal method	Mean 31.1% (± 26.7)	82% of MVR (mech); 54% of patients with MVR (prosthetic)	10.4% of pts (TTR ≥ 70%)	NA	NA	NA
Sana et al., 2020 [43]	Rosendaal method	Mean 48.87 ± 28.69% in 341 patients	NA	32.50	5.80	5.80	1.64
Lauren et al., 2019 [35]	Rosendaal and Direct Method	Mean 29.4% by Rosendaal method and 25.2% by direct method	NA	10	NA	NA	NA
Sonuga et al., 2016 [37]	Cross-section-of-the-files method	48.50%	48	NA	14.00	NA	2.20
Fenta et al., 2017 [23]	Direct Method	Mean 29%	NA	NA	NA	NA	NA
Semakula et al., 2020 [5]	Rosendaal method	Median 41%	NA	NA	NA	NA	NA
Prinsloo et al., 2021 [38]	Rosendaal method	Median 37.2%	NA	17.80%	NA	NA	NA
Ahmed et al., 2017 [41]	Direct Method	BI mean 51.5% and AI 68.3%	BI mean 51.5% and AI 68.3%	NA	BI 37% of patients, AI 53 of patients	0.00	NA
Botsile et al., 2020 [39]	Rosendaal method	Median 29.8%	NA	14.80	14.10	14.10	22.50
Masresha et al., 2021 [31]	Rosendaal method	Mean 41%	NA	29.20	4.50	NA	7.40
Kizito et al., 2016 [47]	Cross-section-of-the-files method	Mean 43.5%	43.50	NA	NA	NA	NA
Yimer et al., 2021[40]	Rosendaal method	Mean 42.03%	NA	12.67	20.67	NA	NA
Sadhabiriss and Brown, 2021 [33]	Rosendaal method	Mean TTR for the AF group was 44.5% and for PHV was 13.7%	NA	10.4% for the AF group had a range of more than 70% but none in the PHV group achieved this	24.00	NA	NA
Ntlokotsi et al., 2018 [28]	Direct method	49.70%	NA	Cut-off TTR was ≥ 70%	0.006% per patient-year	NA	0.002% per patient-year
Rejeb et al., 2019 [29]	Rosendaal method	Mean 57.3%	NA	24.5%; Cut-off TTR was ≥ 70%	0.59	9.50	15 patients
Mwita et al.,2017 [30]	Rosendaal method	Median 30.8%	NA	14.90	NA	NA	NA
Ebrahim et al., 2018 [7]	Rosendaal method	Mean 47%	NA	25.10	NA	NA	NA

*MVR* Mitral valve replacement, *PHV* Prosthetic heart valve, *BI* Before the intervention, *AI* After the intervention, *TTR* Time in therapeutic range, *NA* Not applicable

### Secondary outcomes

Bleeding/hemorrhagic events were reported in three studies as both major and minor bleeding events [29, 39, 43], and the remaining studies that documented these events reported either of them. The highest percentage of bleeding incidence [(59%, (9.5% major bleeding, 49.5% minor bleeding)] was reported by studies carried out in Tunisia [29] and the lowers incidence (0.006% per patient-year) was reported from Dr. George Mukhari Academic Hospital [28] study in South Africa. During follow-up period, six studies [28, 31, 37, 43, 48, 49] reported that 0.002% per-patient year) [43] to 22.5% [39] of the patients developed thromboembolic events. Thromboembolic complications/events in range of 1.64 to 7.5% were occurred in four remaining studies [29, 31, 43, 50]. All-cause hospital admission during the study period was reported only by two studies with the incidence of 32.5% [38] and (10.4%) before intervention vs 3.7% after intervention) [41], respectively. Emergency department visits and mortality during the study period were reported by studies conducted in Ethiopia and Tunisia in 1.5% [31, 51] and 5.6% [43] of patients, respectively (Table 2).

### Factors associated with optimal anticoagulation in patients receiving warfarin

There were various patients’ sociodemographic and clinical characteristics (age, sex, hospitalization, mortality, disease, and medication-related factors) that contributed to poor TTR, and occurrences of bleeding and thromboembolic events. The most frequently reported factors were the presence of comorbidities (heart failure comorbidity [31, 36, 40, 43], renal dysfunction [36], pulmonary hypertension [7]), taking more than two drugs with warfarin [40], presence of potentially interacting medication with warfarin [31], patients’ socio-demographic profile (age less than 50 years [38], female gender and lower education level [47] and smoking [39]). In addition, hospitalization [38] and frequent INR monitoring [7] were also reported as predictors of poor anticoagulation (lower TTR) in included studies. The detail on these associations and other associations with secondary outcomes is provided in Table 3. Only studies that reported significant association were included in the table.

**Table 3:** Factors associated with poor anticoagulation and other secondary outcomes in long term care in Africa

Authors’ name	Factors associated with poor anticoagulation outcomes (low TTR%)	Factors associated with bleeding events	Factors associated with Thromboembolism events	Factors associated with hospitalization events	Factors associated with mortality during warfarin therapy
Karuri et al., 2019 [36]	CHF, renal dysfunction	NA	NA	NA	NA
Sana et al., 2020 [43]	CHF, and nonvalvular AF type	Hypertension and antiplatelet use	obstructive sleep apnea and higher CHA2DS2VASc score	NA	CHF, and hypertension
Prinsloo et al., 2021 [38]	Patents aged < 50, hospitalization	NA	NA	NA	NA
Ahmed et al., 2017 [41]	Absence of pharmacists’ intervention	NA	NA	clinical pharmacy intervention (-)	NA
Botsile et al., 2020 [39]	NA	Duration of warfarin use, Increased level of education	NA	NA	NA
Masresha et al., 2021 [31]	potential medication interaction, presence of co-morbid conditions	NA	NA	NA	NA
Kizito et al., 2016 [47]	female gender, lower education level	NA	NA	NA	NA
Yimer et al., 2021 [40]	Receiving > 2 drugs with warfarin, heart failure comorbidity	NA	NA	NA	NA
Rejeb et al., 2019 [29]	NA	Poor TTR (< 50%)	NA	NA	NA
Mwita et al.,2017 [30]	Smoking and pulmonary hypertension	NA	NA	NA	NA
Ebrahim et al., 2018 [7]	Frequent INR monitoring	NA	NA	NA	NA

*CHF* heart failure, *NA* Not applicable, *TTR* Time in therapeutic range, *AF* Atrial fibrillation, *INR* International normalized ratio

## Discussion

This systematic review was conducted to assess the level of anticoagulation control, treatment outcome, and associated factors among patients receiving warfarin in long-term care in Africa. Suboptimal anticoagulation was reported in this review with TTR ranging from 13.7% to 57.3% as compared to the recommended TTR level (≥ 65%) [52] or ESC 2020TTR recommendation (≥ 70%) [16].

The lowest TTR level was observed in studies conducted in China (38.2%) [21], Lithuania (40%) [53], and Turkey (42.3%) [54]. On the other hand, a higher TTR values of 61.5% [52] and 65% [55] were reported by the FANTASIIA and ORBIT-A registries, respectively. Moreover, a huge variation in the percentage of TTRs was observed in patients receiving warfarin in different African countries. Similarly, TTR variation was seen among different studies conducted in Canada (TTR of 44.2 to 61%) [20, 56, 57], Saudi Arabia, Iran, Kuwait, and Brazil with the mean TTR of 52.6 to 59% [13, 58–60]. However, TTRs reported in this systematic review were lower as compared with reports from Canada (58.76%) [20], the USA (overall mean and median TTR of 65 ± 20% and 68% [IQR 53–79%) and South Africa (58.1% ± 16%) [20, 61, 62]. The discrepancies might be due to the difference in method used to determine TTR, and sample size [14].

Higher TTR is the best indicator of good anticoagulation management service [63]. The lower TTR reported in Africa questioned the quality of anticoagulation service [2, 34]. Despite the presence of several risk factors, this might be partly explained by the limited and ineffective implementation of evidence-based AMS recommended by international guidelines. This includes the inappropriateness of the current setup for providing expected AMS (poorly developed structure in Africa), unavailability of working manuals e.g., functional protocols; resources (coagulation tests and anticoagulants); prescribing anticoagulation prescription with little or no monitoring. absence of specialty anticoagulation clinics/services; lack of a multidisciplinary team in managing anticoagulation service in health facilities [2]. Application of evidence-based strategies should be settled, like implementing ‘warfarin care bundles’ that include process- and patient-centered activities [64], employing interventions that improve INR control [41, 65], decentralization of anticoagulation services, setting up of anticoagulation clinics, improving access to warfarin, improving access to laboratory testing and/or scaling up point-of-care INR testing, task-shifting of anticoagulation care to mid-level health care workers, staff training, and implementing locally validated dose initiation and dose adjustment algorithms [23].

Regarding patients with optimal anticoagulation (i.e., TTR ≥ 65%), a lower percentage of patients (10 to 32.25%) achieved this target. The maximum percentage (32.25%) was reported by Tunisia prospective study [43]. In the same way, the Lithuanian (20%) [53] and Brazilian studies (31%) [60] studies reported a similar range of patients who achieved TTR above 65%. However, a study that evaluated the TTRs in four European countries in AF patients found that 44.2 to 47.8% of patients achieved TTR above 70% and with a higher percentage (65.4%) in United Kingdom patients [66]. A higher percentage of patients with optimal anticoagulation was also reported in Canada [19]. A lower percentage of patients in achieving recommended TTR may indicate a higher likelihood of suboptimal anticoagulation with warfarin in Africa countries which mandate a significant room for improvement of anticoagulation control in countries across low-income countries including Africa. Decentralization of anticoagulation care, together with expanded access to anticoagulants and monitoring, and enhanced support to practitioners and patients, developing and using initiation and maintenance/adjustment dosing protocols that developed by taking consideration of locally relevant factors into account is crucial to achieve better anticoagulation control in resource-limited settings [64].

Our review also explored factors associated with poor anticoagulation in patients receiving warfarin therapy. Having heart failure, renal dysfunction, and pulmonary hypertension comorbidities, taking more than two drugs along with warfarin, presence of interacting medication with warfarin, different socio-demographic characteristics, history of hospitalization, and frequent INR monitoring were identified as predictors of poor anticoagulation. A plethora of literature showed controversial results on the association of age with poor TTR. This review study conducted by Prinsloo et al., in South Africa, showed patients less than 50 years had worsened INR control [38]. A Swedish study reported this correlation the other way round that is the presence of correlation between improved TTR and older age [67]. However, the quality of anticoagulation was minimal in the aged population, and there was a negative association between age and TTR levels in a study conducted in Turkey [54].

Having congestive heart failure as a comorbidity was reported as an independent predictor of poor control of anticoagulation in three studies included in this review [36, 40, 43]. This effect was also documented in patients with non-valvular atrial fibrillation in a private setting in Brazil among patients with atrial fibrillation, and in, Israel [68] among patients with non-valvular atrial fibrillation in primary care (Fantas-TIC Study) [69]. This might be due to abnormal blood flow in patients with left ventricular dysfunction (including regional areas of dyskinesis or aneurysm) resulted in the development of LV thrombus. While all the components of Virchow’s triad may apply to HF patients, blood flow abnormalities are presumed to play the biggest role in imparting stroke risk [70]. This implies that having heart failure may be considered a double burden in managing/ controlling anticoagulation in these patient populations. Furthermore, patients with comorbidities require more drugs/polypharmacy for their management, which makes them more vulnerable to warfarin drug interactions which in turn, affect optimal anticoagulation [31, 40].

### Strength and limitation of study

This systematic review is the first to show anticoagulation control and outcome in different African countries by characterizing time in therapeutic range and other secondary outcomes. The review has some limitations. First, we included only articles reporting in the English language, which may result in the loss of some important studies and thereby underestimation of the findings. Second, the practice of AMS varies across the studies, which require further assessment of TTR pooled estimates. Third, some relevant data (e.g., the incidence of thrombotic and bleeding events) were not reported in most of the studies. Finally, the results of this systematic review may not be representative of all Africa countries as there might be studies that were not included and also due to a limited aspect of care provided in these regions.

## Conclusion and recommendations

Oral anticoagulation control was suboptimal in patients taking warfarin in Africa as evidenced by low TTR when compared with the recommended target by different international guidelines to achieve optimal anticoagulation. Special emphasis should be given to improving AMS in Africa region by working towards optimizing anticoagulation and decreasing harms (thromboembolic and bleeding events) in patients taking anticoagulation. Moreover, establishing dedicated anticoagulation clinics led by pharmacists or multidisciplinary teams using standardized approaches in Africa health care settings may achieve better anticoagulation control than routine models of care, where anticoagulation patients are seen as part of the general patient mix.

## Supplementary Information

**Additional file 1.**

## Data Availability

Not applicable.

## Abbreviations

AF: Atrial fibrillation
AJOL: African Journal of Online
ESC: European Society of Cardiology
INR: International normalized ratio
MESH: Medical Subject Heading
PRISMA: Preferred Reporting Items for Systematic Reviews and Meta-analysis
PROSPERO: Prospective Register of Systematic Reviews
TTR: Time in therapeutic range
VKAs: Vitamin K antagonists

## References

[1] Singer DE, Hellkamp AS, Piccini JP, Mahaffey KW, Lokhnygina Y, Pan G (2013). Impact of global geographic region on time in therapeutic range on warfarin anticoagulant therapy: data from the ROCKET AF clinical trial. J Am Heart Assoc.

[2] Anakwue R (2020). Anticoagulation in sub-saharan africa with the advent of non-vitamin K antagonist oral anticoagulants. Niger J Med.

[3] Jones AE, King JB, Kim K, Witt DM (2020). The role of clinical pharmacy anticoagulation services in direct oral anticoagulant monitoring. J Thromb Thrombolysis.

[4] Nyamu DG, Guantai AN, Osanjo GO, Godman B, Aklillu E (2020). Profiles of patients on warfarin anticoagulation therapy in a leading tertiary referral hospital in Kenya; findings and implications for Kenya. Expert Rev Cardiovasc Ther.

[5] Semakula JR, Mouton JP, Jorgensen A, Hutchinson C, Allie S, Semakula L (2020). A cross-sectional evaluation of five warfarin anticoagulation services in Uganda and South Africa. PLoS ONE.

[6] Mansur AP, Takada JY, Avakian SD, Strunz CMC (2012). Warfarin doses for anticoagulation therapy in elderly patients with chronic atrial fibrillation. Clinics.

[7] Ebrahim I, Bryer A, Cohen K, Mouton JP, Msemburi W, Blockman M (2018). Poor anticoagulation control in patients taking warfarin at a tertiary and district-level prothrombin clinic in Cape Town. S Afr Med J.

[8] Laäs DJ, Naidoo M (2018). An evaluation of warfarin use at an urban district-level hospital in Kwazulu-natal Province. S Afri Med J.

[9] Hirsh J, Guyatt GH (2008). Executive summary: American College of chest physicians evidence-based clinical practice guidelines.

[10] Minno AD, Frigerio B, Spadarella G, Sansaro D, Amato M, Kitzmiller JP (2017). Old and new oral anticoagulants: food, herbal medicines and drug interactions. Blood Rev.

[11] Alghadeeer S, Alzahrani AA, Alalayet WY, Alkharashi AA, Alarifi MN (2020). Anticoagulation control of warfarin in pharmacist-led clinics versus physician-led clinics: a prospective observational study. Risk Manag Healthc Policy.

[12] de Barros e Silva PGM, Sznejder H, Vasconcellos R, Charles GM, Mendonca-Filho HTF, Mardekian J, et al. Anticoagulation therapy in patients with non-valvular atrial fibrillation in a private setting in Brazil: A real-world study. Arq Bras Cardiol. 2020;114(3):457–66. 10.36660/abc.20180076 PMC7792730 32049154

[13] Farsad B, Abbasinazari M, Dabagh A, Bakshandeh H (2016). Evaluation of time in therapeutic range in patients with non-valvular atrial fibrillation receiving treatment with warfarin in Tehran Iran: a cross-sectional study. J Clin Diagn Res.

[14] Pharmd LS, Speckman J, Ansell J (2003). Quality assessment of anticoagulation dose management: comparative evaluation of measures of time-in-therapeutic range. J Thromb Thrombolysis.

[15] Pastori D, Pignatelli P, Saliola M, Carnevale R, Vicario T, Del M (2015). Inadequate anticoagulation by Vitamin K Antagonists is associated with Major Adverse Cardiovascular Events in patients with atrial fi brillation. Int J Cardiol.

[16] Hindricks G, Potpara T, Dagres N, Bax JJ, Boriani G, Dan GA (2021). 2020 ESC Guidelines for the diagnosis and management of atrial fibrillation developed in collaboration with the European Association for Cardio-Thoracic Surgery (EACTS). Eur Heart J.

[17] Lip GYH (2015). Stroke prevention in atrial fibrillation: changing concepts. Eur Heart J.

[18] Baker JW, Pierce KL, Ryals CA (2011). INR goal attainment and oral anticoagulation knowledge of patients enrolled in an anticoagulation clinic in a veterans affairs medical center. J Manag Care Pharm.

[19] Caldeira D, Cruz I, Morgado G, Stuart B, Gomes C, Martins C (2014). Evaluation of time in therapeutic range in anticoagulated patients : a single-center, retrospective, observational study. BMC Res Notes.

[20] Gateman D, Trojnar ME, Agarwal G (2017). Time in therapeutic range Un RIN dans la fourchette thérapeutique. Can Fam Physician..

[21] Chan P, Li WH, Hai J, Chan EW, Wong ICK, Tse H, et al. Time in therapeutic range and percentage of INRs in therapeutic range as measure of quality of anticoagulation control in atrial fibrillation patients. Can J Cardiol. 2015. 10.1016/j.cjca.2015.10.029 10.1016/j.cjca.2015.10.029 26927855

[22] Han SY, Palmeri ST, Broderick SH, Hasselblad V, Rendall D, Stevens S (2013). Quality of anticoagulation with warfarin in patients with nonvalvular atrial fibrillation in the community setting. J Electrocardiol.

[23] Fenta TG, Assefa T, Alemayehu B (2017). Quality of anticoagulation management with warfarin among outpatients in a tertiary hospital in Addis Ababa Ethiopia: a retrospective cross-sectional study. BMC Health Serv Res.

[24] Page MJ, McKenzie JE, Bossuyt PM, Boutron I, Hoffmann TC, Mulrow CD, The PRISMA (2020). Statement: an updated guideline for reporting systematic reviews. BMJ.

[25] Harrison H, Griffin SJ, Kuhn I, Usher-smith JA (2020). Software tools to support title and abstract screening for systematic reviews in healthcare : an evaluation. BMC Med Res Methodol.

[26] Moola S, Munn Z, Tufanaru C, Aromataris E, Sears K, Sfetcu R, Currie M, Qureshi R, Mattis P, Lisy K MP-F. Checklist for analytical cross sectional studies. Joanna Briggs Inst Rev Man. 2017;1–7. Available from: http://joannabriggs.org/research/critical-appraisal-tools.

[27] Schulman S, Anger SU, Bergqvist D, Eriksson B, Lassen MR, Fisher W (2010). Definition of major bleeding in clinical investigations of antihemostatic medicinal products in surgical patients. J Thromb Haemost.

[28] Ntlokotsi S, Moshesh MF, Mntla P, Towobola OA, Mogale MA (2018). Optimum INR intensity and therapeutic INR control in patients with mechanical heart valve prosthesis on warfarin oral anticoagulation at Dr George Mukhari academic hospital: a three-year retrospective study. South African Fam Pract.

[29] Ben RO, Brahim W, Ghali H, Ernez S, Mahdhaoui A, Jeridi G (2019). Epidemiology of thromboembolic and hemorrhagic events in patients with atrial fibrillation under anti-vitamin K. Tunis Med.

[30] Mwita JC, Francis JM, Oyekunle AA, Gaenamong M, Goepamang M, Magafu MGMD (2018). Quality of anticoagulation with warfarin at a Tertiary Hospital in Botswana. Clin Appl Thromb Hemost.

[31] Masresha N, Muche EA, Atnafu A, Abdela O (2021). Evaluation of warfarin anticoagulation at university of Gondar comprehensive specialized hospital, north-west Ethiopia. J Blood Med.

[32] Abusin S (2019). Using whatsapp smartphone application to monitor INR in patients on warfarin: first experience with 21 patients. Sudan Hear J.

[33] Sadhabariss D, Brown SL (2021). Warfarin: time in therapeutic range, a single centre study on patients using warfarin for stroke prevention in non-valvular atrial fibrillation and prosthetic heart valves. SA Hear.

[34] Semakula JR, Kisa G, Mouton JP, Cohen K, Blockman M, Pirmohamed M (2021). Anticoagulation in sub-Saharan Africa: are direct oral anticoagulants the answer? A review of lessons learnt from warfarin. Br J Clin Pharmacol..

[35] Jonkman LJ, Gwanyanya MP, Kakololo MN, Verbeeck RK, Singu BS (2019). Assessment of anticoagulation management in outpatients attending a warfarin clinic in Windhoek. Namibia. Drugs Ther Perspect..

[36] Karuri S, Nyamu D, Opanga S, Menge T. Factors associated with time in therapeutic range among patients on oral anticoagulation therapy in a tertiary teaching and referral hospital in Kenya. East Cent African J Pharm Sci. 2019;22(3):85–95. Available from: http://uonjournals.uonbi.ac.ke/ojs/index.php/ecajps/article/view/293

[37] Sonuga BO, Hellenberg DA, Cupido CS, Jaeger C (2016). Profile and anticoagulation outcomes of patients on warfarin therapy in an urban hospital in Cape town, South Africa. African J Prim Heal care Fam Med.

[38] Prinsloo DN, Gould TJ, Viljoen CA, Basera W, Ntsekhe M (2021). International normalised ratio control in a non-metropolitan setting in Western Cape Province. S Afr Med J.

[39] Botsile E, Mwita JC (2020). Incidence and risk factors for thromboembolism and major bleeding in patients with mechanical heart valves: a tertiary hospital-based study in Botswana. Cardiovasc J Afr.

[40] Yimer NS, Abiye AA, Hussen SU, Tadesse TA (2021). Anticoagulation Control, Outcomes, and Associated Factors in Patients with Atrial Fibrillation Receiving Warfarin at Tertiary Care Hospital in Ethiopia. Clin Appl Thromb..

[41] Ahmed NO, Osman B, Abdelhai YM, El-Hadiyah TMH (2017). Impact of clinical pharmacist intervention in anticoagulation clinic in Sudan. Int J Clin Pharm.

[42] Mariita K, Maina C, Nyamu D, Menge T, Karimi P. Patient factors impacting on oral anticoagulation therapy among adult outpatients in a Kenyan referral hospital. African J Pharmacol Ther. 2016;5(3):193–200. Available from: http://journals.uonbi.ac.ke/ajpt/article/view/1534

[43] Ouali S, Ben Halima A, Chabrak S, Chettaoui R, Ben Halima M, Haggui A (2021). Epidemiological characteristics, management, and outcomes of atrial fibrillation in TUNISIA: Results from the National Tunisian Registry of Atrial Fibrillation (NATURE-AF). Clin Cardiol.

[44] Mwita JC, Francis JM, Oyekunle AA, Gaenamong M, Goepamang M, Magafu MGMD (2018). Quality of anticoagulation with warfarin at a tertiary hospital in Botswana.

[45] Ouali S, Mechri M, Ali Z Ben, Boudiche S, Halima M Ben, Rejaibi S, et al. Les facteurs associés à la qualité de l ’ anticoagulation chez les patients sous antivitamines K en Tunisie Factors associated to adequate time in therapeutic range with oral vitamin K antagonists in Tunisia. Tunis Med. 2016;97(01):1–9. 31535702

[46] Schapkaitz E, Jacobson BF, Becker P, Conway G (2006). Thrombo-embolic and bleeding complications in patients with mechanical valve replacements–a prospective observational study. S Afr Med J.

[47] Mariita K, Nyamu DG, Maina CK, Karimi PN (2016). Patient factors impacting on oral anticoagulation therapy among adult outpatients in a Kenyan referral hospital. Afr J Pharmacol Ther..

[48] Botsile E, Mwita JC (2020). Cardiovascular Topics Incidence and risk factors for thromboembolism and major bleeding in patients with mechanical heart valves : a tertiary hospital-based study in Botswana.

[49] Ouali S, Ben Halima A, Chabrak S, Chettaoui R, Ben Halima M, Haggui A (2021). Epidemiological characteristics, management, and outcomes of atrial fibrillation in TUNISIA: Results from the National Tunisian Registry of Atrial Fibrillation (NATURE-AF). Clin Cardiol.

[50] Sonuga BO, Hellenberg DA, Cupido CS, Jaeger C, Hospital V, Sonuga B (2016). Profile and anticoagulation outcomes of patients on warfarin therapy in an urban hospital in Cape Town, South Africa. Afr J Prim Health Care Fam Med.

[51] Masresha N, Muche EA, Atnafu A, Abdela O (2021). Evaluation of warfarin anticoagulation at University of Gondar Comprehensive Specialized. J Blood Med.

[52] Esteve-Pastor MA, Rivera-Caravaca JM, Roldán-Rabadán I, Roldán V, Muñiz J, Raña-Míguez P (2018). Quality of oral anticoagulation with Vitamin K antagonists in “real-world” patients with atrial fibrillation: a report from the prospective multicentre FANTASIIA registry. Europace.

[53] Urbonas G, Valius L, Šakalytė G, Petniūnas K, Petniūnienė I (2019). The quality of anticoagulation therapy among warfarin-treated patients with atrial fibrillation in a primary health care setting. Med.

[54] Ugur A, Turk O, Tuncer E, Alioglu E, Yuksel K (2015). Evaluation of the impact of warfarin ’ s time-in-therapeutic range on outcomes of patients with atrial fibrillation in Turkey : perspectives from the observational, prospective WATER registry.

[55] Pokorney SD, Holmes DN, Thomas L, Fonarow GC, Kowey PR, Reiffel JA (2019). Association between Warfarin Control Metrics and Atrial Fibrillation Outcomes in the Outcomes Registry for Better Informed Treatment of Atrial Fibrillation. JAMA Cardiol.

[56] Quinn LM, Richardson R, Cameron KJ, Battistella M (2015). Evaluating time in therapeutic range for hemodialysis patients taking warfarin. Clin Nephrol.

[57] Defoe K, Wichart J, Leung K (2021). Time in therapeutic range using a nomogram for dose adjustment of warfarin in patients on hemodialysis with atrial fibrillation. Can J Kidney Heal Dis..

[58] Alyousif SM, Alsaileek AA. Quality of anticoagulation control among patients with atrial fibrillation : An experience of a tertiary care center in Saudi Arabia. J Saudi Hear Assoc. 2016;1–5. 10.1016/j.jsha.2016.02.001 10.1016/j.jsha.2016.02.001 PMC5034360 27688671

[59] Zubaid M, Saad H, Ridha M, Nair KKM, Rashed W, Alhamdan R (2013). Quality of anticoagulation with warfarin across Kuwait. Hell J Cardiol..

[60] Carvalho AR, Ciol MA, Tiu F, Rossi LA, Dantas RAS (2013). Anticoagulação oral: Impacto da terapia na qualidade de vida relacionada à saúde ao longo de seis meses. Rev Lat Am Enfermagem.

[61] Pokorney SD, Ms DNS, Thomas L, Fonarow GC, Kowey PR, Chang P, et al. Outcomes registry for better informed treatment of atrial fibrillation Investigators. Am Heart J. 2015. 10.1016/j.ahj.2015.03.017

[62] Parbhoo P, Jacobson B (2019). Articles A Comparison between TTR and FIR As a Measure of the Quality of Anticoagulation in Patients with Atrial Fibrillation. Wits J Clin Med..

[63] Pastori D, Farcomeni A, Saliola M, Del F, Pignatelli P. European Journal of Internal Medicine Temporal trends of time in therapeutic range and incidence of cardiovascular events in patients with non-valvular atrial fi brillation. Eur J Intern Med. 2018;(January):0–1. 10.1016/j.ejim.2018.04.007 10.1016/j.ejim.2018.04.007 29655807

[64] Mouton JP, Blockman M, Sekaggya-Wiltshire C, Semakula J, Waitt C, Pirmohamed M (2021). Improving anticoagulation in sub-Saharan Africa: What are the challenges and how can we overcome them?. Br J Clin Pharmacol..

[65] Nyamu DG, Guantai AN, Osanjo GO, Gitonga I, Kanyiri ML (2017). Predictors of adequate ambulatory anticoagulation among adult patients in a tertiary teaching and referral hospital in Kenya. Afr J Pharmacol Ther..

[66] Benhaddi H, Duprat-lomon I, Doble A, Marchant N, Letierce A, Huguet M (2014). Vitamin K Antagonist Treatment in Patients With Atrial Fibrillation and Time in Therapeutic Range in Four European Countries. Clin Ther..

[67] Wieloch M, Sjlander A, Frykman V, Rosenqvist M, Eriksson N, Svensson PJ (2011). Anticoagulation control in Sweden: Reports of time in therapeutic range, major bleeding, and thrombo-embolic complications from the national quality registry AuriculA. Eur Heart J..

[68] Melamed OC, Horowitz G, Elhayany A, Vinker S (2011). Quality of anticoagulation control among patients with atrial fibrillation. Am J Manag Care..

[69] Llorca MRD, Aguilar C, Carrasco-querol N, Hern Z, Drago EF, Rodr D (2021). Anticoagulation control with acenocoumarol or warfarin in non-valvular atrial fibrillation in primary care (Fantas-TIC Study). Int J Environ Res Public Health..

[70] Zeitler EP, Eapen ZJ, Clinical D, Nc D. Anticoagulation in Heart Failure : a Review. J Atr Fibrillation. 2015;8(1):31–8. 10.4022/jafib.1250 PMC4682916 27957180

