# Supplementary material for: Anticoagulation control, outcomes, and associated factors in long-term-care patients receiving warfarin in Africa: a systematic review
Source: Thromb J. 2022 Oct 3;20:58. doi: 10.1186/s12959-022-00416-9 (PMC9528137; doi:10.1186/s12959-022-00416-9)
Supplement: Supplementary file 1 — Additional file 1. [file 12959_2022_416_MOESM1_ESM.docx]

**Quality assessment of the included studies based on modified JBI’s critical appraisal for cross-sectional studies**

| **Author ID** | **Joanna Briggs Institute’s critical appraisal checklist** | | | | | | | | | |
| --- | --- | --- | --- | --- | --- | --- | --- | --- | --- | --- |
|  | **Q1** | **Q2** | **Q3** | **Q4** | **Q5** | **Q6** | **Q7** | **Q8** | **Total** | **%** |
| Salaheldin 2019 | No | No | No | No | No | No | Yes | No | 1 | 12.5 |
| Karuri 2019 | Yes | Yes | Yes | Yes | Yes | Yes | Yes | Yes | 8 | 100 |
| Sana 2020 | Yes | Yes | Yes | Yes | Yes | Yes | Yes | Yes | 8 | 100 |
| Lauren 2019 | Yes | Yes | Yes | Yes | No | No | Yes | Yes | 6 | 75 |
| Sonuga 2016 | Yes | Yes | Yes | Yes | No | No | Yes | Yes | 6 | 75 |
| Fenta 2017 | Yes | No | Yes | Yes | No | No | Yes | Yes | 5 | 62.5 |
| Semakula2020 | No | No | No | No | No | No | Yes | No | 1 | 12.5 |
| Prinsloo 2021 | Yes | No | No | Yes | Yes | Yes | Yes | Yes | 6 | 75 |
| Ahmed 2017 | Yes | No | Yes | Yes | No | No | No | No | 5 | 62.5 |
| Botsile 2020 | Yes | Yes | No | Yes | Yes | Yes | Yes | Yes | 7 | 87.5 |
| Masresha 2021 | Yes | Yes | Yes | Yes | Yes | Yes | No | Yes | 7 | 87.5 |
| Kizito 2016 | Yes | Yes | No | No | Yes | Yes | No | Yes | 5 | 62.5 |
| Yimer 2021 | Yes | Yes | Yes | Yes | Yes | Yes | Yes | Yes | 8 | 100 |
| Sadhabiriss 2021 | Yes | Yes | Yes | Yes | Yes | Yes | Yes | Yes | 8 | 100 |
| Ntlokotsi, 2018 | Yes | Yes | Yes | Yes | Yes | Yes | Yes | Yes | 8 | 100 |
| Rejeb 2019 | Yes | Yes | No | Yes | No | No | Yes | Yes | 6 | 75 |
| Mwita 2017 | No | Yes | Yes | No | Yes | Yes | Yes | Yes | 7 | 87.5 |
| Ebrahim 2018 | Yes | Yes | Yes | Yes | Yes | Yes | Yes | Yes | 8 | 100 |

[Q1-8, JBI’s Critical Appraisal Checklist for Analytical Cross-Sectional studies [Q1: Were the criteria for inclusion in the sample clearly defined? Q2: Were the study subjects and the setting described in detail? Q3: Was the exposure measured in a valid and reliable way? Q4: Were objective, standard criteria used for measurement of the condition? Q5: Were confounding factors identified? Q6: Were strategies to deal with confounding factors stated? Q7: Were the outcomes measured in a valid and reliable way? Q8: Was an appropriate statistical analysis used?]
